# Genome-wide changes accompanying the knockdown of Ep-CAM in retinoblastoma

**Published:** 2010-05-11

**Authors:** Moutushy Mitra, Mallikarjuna Kandalam, Rama Shanker Verma, Krishnan UmaMaheswari, Subramanian Krishnakumar

**Affiliations:** 1 Department of Ocular Pathology, Vision Research Foundation, Sankara Nethralaya, Chennai, India; 2 Department of Biotechnology, Indian Institute of Technology, Madras, India; 3 CeNTAB SASTRA University, Tanjore, India

## Abstract

**Purpose:**

Previously we showed that epithelial cell adhesion molecule (Ep-CAM), a cell surface molecule, was highly expressed in primary retinoblastoma tumors. In the present study, we studied the genes regulated by Ep-CAM in a retinoblastoma Y79 cell line in vitro using a combination of short interference RNA and microarray technology.

**Methods:**

Flow cytometry, quantitative reverse transcriptase PCR (Q-RT–PCR), and immunohistochemistry were performed to confirm the Ep-CAM re-expression in the Y79 cells treated with 5′-azacytidine (AZC). Ep-CAM expression in AZC-treated Y79 cells was silenced using synthetic anti-Ep-CAM short interference RNA, and whole genome microarray was performed to determine the gene expression changes post Ep-CAM knockdown. Ep-CAM inhibition was confirmed by Q-RT–PCR, western blotting, and immunofluorescence.

**Results:**

Ep-CAM expression was significantly restored in Y79 cells on day 5 of AZC treatment. Ep-CAM inhibition significantly affected Y79 cell proliferation. We identified 465 upregulated genes (≥1.0 fold) and 205 downregulated genes (≤0.5 fold) in response to knockdown of Ep-CAM. These genes regulate several aspects of tumor function, including cell survival/proliferation, DNA replication/transcription, apoptosis, and angiogenesis. Quantitative pathway analysis using Biointerpreter further revealed that the most pronounced effect of Ep-CAM knockdown was deregulation of pathways that include mitogen-activated protein kinase (MAP) kinase and tumor protein 53 (P53) pathways. Real-time Q-RT–PCR confirmed microarray gene expression changes for selected genes.

**Conclusions:**

Ep-CAM silencing significantly decreases Y79 cell proliferation and revealed a wide network of deregulated pathways in vitro. Future studies targeting Ep-CAM gene expression in vivo will help to delineate the mechanisms associated with Ep-CAM gene function in neoplastic transformation and define the potential for Ep-CAM-based molecular intervention in retinoblastoma patients.

## Introduction

Retinoblastoma (RB) is the most common intraocular malignancy in children [1]. For many years retinoblastoma confined to the eye has been a curable disease with local therapy, such as enucleation, external beam irradiation, brachytherapy, cryotherapy, or laser coagulation [2]. In contrast, systemic disease is difficult to cure, although it usually responds to chemotherapy [3–5]. The advent of short interfering (si)RNA might prove a useful addition to, or a substitute for, conventional treatment modalities. Previously we demonstrated the expression of epithelial cell adhesion molecule (Ep-CAM) in RB tumor tissues, and the tumors with choroidal invasion/optic nerve invasion showed significantly higher expression of Ep-CAM [6]. Ep-CAM is a 40,000 MW type I transmembrane glycoprotein that consists of two epidermal growth factor-like extracellular domains, a cysteine-poor region, a transmembrane domain, and a short cytoplasmic tail. Ep-CAM is overexpressed in various epithelial cancers [7] and is an ideal therapeutic target because of the following reasons: (a) overexpression in cancer cells versus noncancerous cells, (b) apical expression in cancer cells and basolateral expression in normal epithelial cells [8], and (c) not shed into the circulation [9]. For these reasons Ep-CAM has gained interest as a potential therapeutic target and an attractive candidate tumor-associated antigen to serve as a target for antibody-based immunotherapy [10,11]. There is evidence that Ep-CAM expression levels correlate with proliferative activity and contribute to neoplastic transformation [12,13]. These data suggest that Ep-CAM is a potential target for molecular intervention and that it requires further investigation.

To define the mechanisms associated with Ep-CAM gene silencing, we investigated the effect of Ep-CAM siRNA treatment on the whole genome expression by microarray technology.

## Methods

### Cell lines and cell culture

Y79 was obtained from the American Type Culture Collection (Manassas, VA). Media and fetal bovine serum (FBS) were purchased from Gibco-BRL (Rockville, MD). Y79 was cultured in Rosewell Park Memorial Institute (RPMI; Gibco-BRL) 1640 supplemented with 10% heat-inactivated fetal calf serum, 0.1% ciprofloxacin, 2 mM L-glutamine, 1 mM sodium pyruvate, and 4.5% dextrose and grown in suspension at 37 °C in a 5% CO_2_-humidified incubator. The study adhered to the Declaration of Helsinki. This study was conducted at the Medical Research Foundation and Vision Research Foundation, Sankara Nethralaya, India, and was approved by the Vision Research Foundation ethics boards.

### Re-expression of epithelial cell adhesion molecule by 5′-azacytidine

Approximately 1×10^5^ Y79 cells were incubated in culture medium with and without 5′-azacytidine (AZC) at a final concentration of 5 µM, with medium changes every day for 5 days. After day 5, the Y79 cells were withdrawn from AZC exposure and subsequently passaged for 30 days in complete medium. Cells were harvested on days 5, 8, 10, 15, and 20 for extraction of total RNA and were tested for Ep-CAM gene expression, using real-time PCR. Also, 1x10^5^ cells were harvested on days 5, 8, 10, and 15 and analyzed for Ep-CAM protein expression, using fluorescence-activated cell sorting.

### RNA interference

Gene silencing of Ep-CAM expression was performed essentially as described previously using sequence-specific siRNA and transfection reagents [14]. For each well of cells, 200 nm of siRNA and 12 µl of Hi-perfect transfection reagent was mixed with 88 µl of serum free RPMI medium in a vial and incubated for 5–10 min at room temperature (RT) to allow the transfection complex formation. Then the complex was added drop-wise to the wells containing Y79 cells and the plate was gently swirled to ensure the uniform distribution of the transfection complex. The plates were incubated further for 6 h in a CO_2_ incubator at 37 °C. After 4 h of incubation, 400 µl of complete RPMI medium was added to the wells and incubated further for 24–72 h. Briefly, 1x10^5^ cells were plated in each well of six-well plates and allowed to grow for 24–36 h (until they were 40%–60% confluent). siRNA was then transfected into cells at a concentration of 200 nM using Hi-Perfect transfection reagent (Qiagen, Santa Clara, CA) and serum-free medium. After 4 h of incubation, serum-rich medium was added. Human Ep-CAM siRNA (Hs_TACSTD1_10; catalog number SI04343416; Forward strand: GGA ACU CAA UGC AUA ACU Att and the reverse strand: UAG UUA UGC AUU GAG UUC Cct) and scrambled siRNA (catalog number 1022563) were used in this study.

### Epithelial cell adhesion molecule relative RNA quantification by real-time quantitative reverse transcriptase PCR

To quantify RNA expression of Ep-CAM in untreated or AZC-treated Y79 cell lines and untreated or siRNA-treated Y79 cells, RNA was extracted by the guanidine isothiocyanate and chloroform method (TRI Reagent; Sigma-Aldrich, Bangalore, India). Cells were harvested from cultures and collected in RNase free vials. To the pellet, one ml of trizol reagent (Sigma-Aldrich) was added and vortexed for 2 min and incubated at room temperature for 5 min. Later, 0.5 ml of chloroform was added to the solution and shook well for 15 s and centrifuged. The aqueous layer that contains RNA was transferred to new vials and 0.5 ml of isopropanol was added and incubated at room temperature for 10 min. After centrifugation, supernatant was discarded and 0.5 ml of 75% ethanol was added and mixed well, centrifuged and supernatant was discarded. Pellet was air dried at RT for 2 min and reconstituted in 25 µl of RNase free water. All the centrifugations in RNA extraction was performed at 15,000× g for 10 min at 4 °C. All RNA samples were treated with DNase (Turbo; Ambion, Genetix Biotech Asia Pvt. Ltd., Chennai, India). For all samples, 1 μg of total RNA was used to synthesize first-strand cDNA with reverse transcriptase (SuperScript II; Invitrogen, Joyvel, Chennai, India) and random primers. The cDNA synthesis was performed at 37 °C for 60 min after heat inactivation at 95 °C for 10 min. Gene expression assays for *Ep-CAM* (Hs00158980_m1) and two endogenous controls, Glyceraldehyde-3-phosphate dehydrogenase (*GAPDH*; Hs99999905_ml) and *HPRT* (Hs99999909_m1), were obtained from Applied Biosystems (LabIndia, Chennai, India). The primer sequences used for Q-RT–PCR are as follows: Homo sapiens proliferating cell nuclear antigen (*PCNA)*: forward primer (FP) 5′- CAG GGC TCC ATC CTC AAG AA-3′, reverse primer (RP) 5′-TCT TCA TTG CCG GCG CAT T-3′; *FOS*: FP 5′-CAG CGA GCA ACT GAG AAG CC-3′, RP 5′-CGC TGT GAA GCA GAG CTG G-3′; *JUN*: FP 5′-CAA AGT TTG GAT TGC ATC AAG TG-3′, RP 5′-TAA CAT TAT AAA TGG TCA CAG CAC ATG-3′; *DRAM*: FP 5′-ATG GTC ATC TCT GCC GTT TC-3′, RP 5′-AAA GGC CAC TGT CCA TTC AC-3′; *CCND3*: FP 5′-TGG ATG CTG GAG GTA TGT G-3′, RP 5′-CGT GGT CGG TGT AGA TGC-3′. Quantification of gene expression was performed in triplicate in a 20 μl volume in 96-well plates on a real-time PCR system (Prism 7300; ABI, Lab India Instruments, Gurgaon, India). Each reaction included 1× primer probe mix (*Taq*Man; ABI), 1× universal PCR master mix (*Taq*Man; ABI), and 100 ng of cDNA. The PCR was performed as follows: 2 min at 50 °C, 10 min at 95 °C, and 40 cycles of 15 s at 95 °C, plus 1 min at 60 °C. Commercial software (SDS ver. 1.3; ABI) was used to calculate ΔΔCt [15] relative expression values for *Ep-CAM* normalized to the *GAPDH* endogenous control.

### Immunocytochemistry

Untreated or siRNA-treated Y79 cells and untreated or AZC-Y79 cells were harvested, washed, and then fixed in 4% paraformaldehyde and processed for immunofluorescence, using anti-Ep-CAM mouse monoclonal antibody (Santa Cruz Biotechnology, Santa Cruz, CA) as the primary antibody at a dilution of 1:100 and incubating for 12 h at 4 °C. Fluorescein isothiocynanate (FITC)-labeled antimouse antibody was used as the secondary antibody. Incubation with the secondary antibody was for 30 min at 37 °C. Immunofluorescence staining was detected using an Axio Observer fluorescent microscope (Carl Zeiss, Berlin, Germany). Due to the lack of purified Ep-CAM protein, we could not use an adsorbed control reaction for the negative control. Instead, we used isotypic antibodies (normal mouse serum) as negative controls, both for untreated and AZC-treated Y79 cells, in the immunohistochemistry. For immunohistochemistry, Novolink Polymer kit (Leica Microsystems Inc., Bannockburn, IL) was used after primary antibody incubation for developing the reaction, and hematoxylin was used for counterstaining. Immunostaining was assessed based on intensity and was graded as 1+ (low), 2+ (moderate), and 3+ (strong).

### Flow cytometric analysis

Y79 cells were harvested, washed, and the cell number determined. Approximately 3x10^5^ cells/ml were resuspended in ice-cold phosphate buffered saline (PBS; 137 mM NaCl, 2.7 mM KCl, 10 mM sodium phosphate dibasic, 2 mM potassium phosphate monobasic and a pH of 7.4), 10% fetal calf serum, and 1% sodium azide. Mouse monoclonal primary antibody against Ep-CAM with a final dilution of 1:40 (C-10; Santa Cruz Biotechnology) was used and incubated for 2 h at 4 °C. After incubation, cells were washed three times (at 400× g for 5 min each) with ice-cold PBS. FITC-conjugated antimouse secondary antibody with a final dilution of 1:1,000 was used and incubated for 1 h at 4 °C in the dark. After incubation, cells were washed three times (centrifugation at 400× g for 5 min each) with ice-cold PBS. Cells were analyzed on a FACSCalibur flow cytometer (BD Biosciences, San Jose, CA), using the CellQuest software program (BD Biosciences). The fluorescence of FITC-stained cells was excited with an argon laser at 488 nm, and the emissions were detected at 530 nm.

### Dimethylthiazol-2-yl)-2,5-diphenyltetrazolium bromide (MTT) test for 5′-azacytidine cytotoxicity and EpCAM siRNA effect

Cultures were set up in triplicate and treated or not with 5 μM AZC for 24, 48, 72, 96, and 120 h. After 5 days of AZC treatment, the cells were washed with PBS and incubated with complete medium for 72 h. Then, siRNA (Qiagen, Santa Clara, CA) was added to the plates, and the cells were allowed to grow for 24, 48, or 72 h. At each time point, cells were checked visually for growth and proliferation. 3-(4,5-Dimethylthiazol-2-yl)-2,5-diphenyltetrazolium bromide (MTT; Sigma, St Louis, MO) was then added to the wells, and the cells were incubated at 37 °C for 4 h. MTT solubilization solution (10% Triton X-100 in acidic isopropanol, 0.1 N HCl) was then added, and the cells were incubated overnight. Colorimetric measurements were made using a spectrophotometer (Beckman Coulter India Private Ltd, New Delhi, India) at 562 nm, and the background was subtracted at 650 nm.

### Western blot analysis

Cells were lysed in radioimmunoprecipitation assay lysis buffer for 15 min on ice. An aliquot (100 µg) of lysate was electrophoresed by 10% sodium dodecyl sulfate-polyacrylamide gel and blotted onto a nitrocellulose membrane. Membranes were blocked in 5% fat-free milk and then incubated separately with 1:500 diluted mouse monoclonal primary antibody against Ep-CAM (C-10) overnight at 4 °C. β-actin was used as a loading control (AC-15, dilution: 1:4,000; Sigma). After washing, membranes were incubated with horseradish peroxidase-conjugated antimouse gamma immunoglobulin (IgG) antibody (diluted to 1:2,000; Santa Cruz Biotechnology) for 1 h at room temperature. The bands were visualized using an enhanced chemiluminescence kit (Amersham, Pittsburgh, PA). Each experiment was performed in triplicate.

### Oligonucleotide arrays

Total RNA used for the microarray analysis was isolated from cultured cells using TRIZOL reagent (Invitrogen, Carlsbad, CA) and treated with TURBO DNase (Ambion, Genetix Biotech Asia Pvt. Ltd., New Delhi, India) to remove the DNA. The RNA samples (10 μg each) in a 50-μl reaction were treated with 1 μl of TURBO DNase (2 U) in 1× TURBO DNase buffer at 37 °C for 30 min. After incubation, the RNA sample was extracted with phenol/chloroform to inactivate TURBO DNase. Agilent's (Agilent Technologies Genotypic, Bangalore, India) Low RNA Input Linear Amplification Kit PLUS was used to generate fluorescent complementary RNA (cRNA). This method uses T7 RNA polymerase, which simultaneously amplifies target material and incorporates cyanine 3-labeled cytidine tri-phosphate (CTP). Qiagen’s RNeasy mini spin columns were used for purification of the amplified cRNA samples, and the samples were then hybridized to the Human Whole Genome 44K Oligo Microarray for 17 h at 65 °C, as recommended by the manufacturer (Agilent Technologies). Data analysis was done using Genespring GX version 10 (Agilent Technologies). Agilent Feature Extraction software (G25677AA; Agilent Technologies) was used to analyze the microarray data.

### Statistical analysis

The statistical analysis included the independent *t* test. Statistical analysis was performed using SPSS version 12.0 software (Chicago, IL). P values less than 0.05 were considered significant.

## Results

### Treatment of Y79 cells with 5′-azacytidine promoted an arrest of cell growth

**Figure 1:**
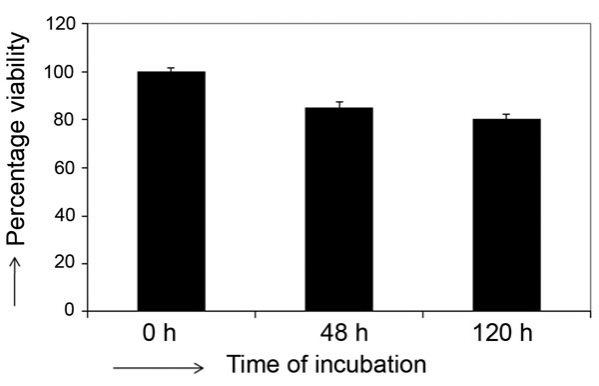
The effect of azacytidine (AZC) treatment on retinoblastoma Y79 cell survival for 5 days was determined by 3-(4,5-dimethylthiazol-2-yl)-2,5-diphenyltetrazolium bromide (MTT assay).The bar diagram shows that 80% percent of Y79 cells were viable on 5th day of AZC treatment at 5 µM concentration. Error bar represents the standard deviation of triplicate values.

Y79 cells were exposed to 5 μM AZC for 24, 48, 72, 96, and 120 h. We first examined whether AZC treatment affected the kinetics of cell growth by evaluating cell viability using the MTT assay. Cell viability kinetics were compared to those of control cells cultured in the absence of AZC. The data of the MTT assay are indicative of both viability and cell proliferation. Treatment with AZC greatly modified these data; around 80% of the number of metabolically active cells were observed on day 5 (Figure 1). This result is consistent with cell cycle arrest in Y79 cells.

### Decreased expression of epithelial cell adhesion molecule in Y79 and its re-expression by 5′-azacytidine treatment

**Figure 2:**
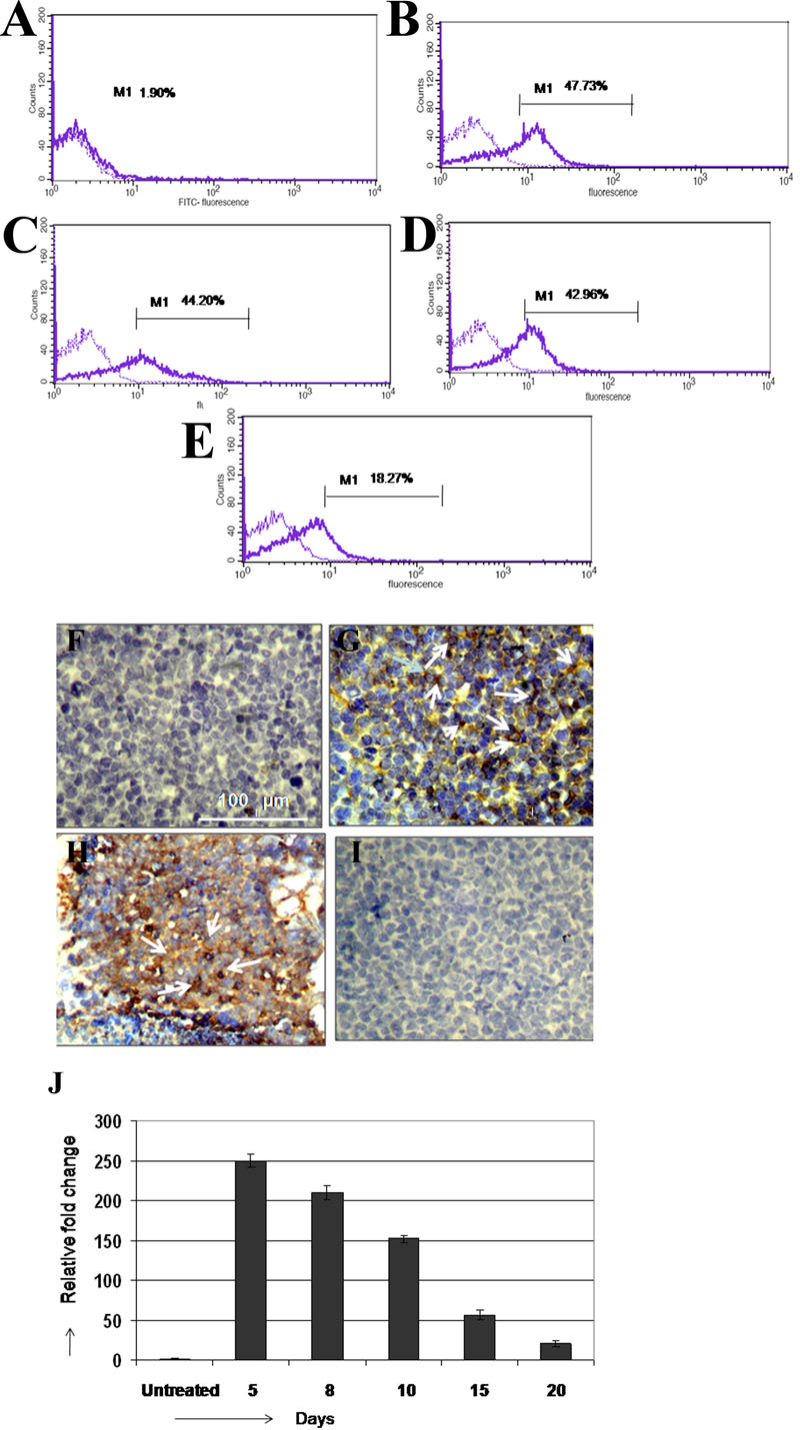
The retinoblastoma Y79 cell line was treated with 5 µM Azacytidine (AZC) and the epithelial cell adhesion molecule (Ep-CAM) expression was studied by flow cytometry analysis (FC), Immunohistochemistry (IHC) and real time quantitative reverse transcriptase polymerase chain reaction (Q-RT–PCR). **A-E**: M1 represents the EpCAM percentage positivity in the FC graphs (solid line). FC analysis demonstrated 47.73%, 44.2%, 42.96%, and 18.27% of Y79 cells positive for Ep-CAM on days 5, 8, 10, and 15 of AZC treatment, respectively. **F-I**: Immunohistchemistry pictures stained with Di-amino benzidine (DAB) and was counter stained with hematoxylin. All the pictures were captured at 20 times magnification. EpCAM was not stained in Y79 cells not treated with AZC (**F**); Strong membrane positive expression of Ep-CAM was seen in Y79 cells treated AZC on 5th day (**G**); Strong membrane positivity of Ep-CAM in the retinoblastoma primary tumor represented as positive control (**H**); Y79 cells were stained negative when isotypic antibodies were used as the negative control (**I**). Q-RT–PCR shows the mRNA expression of Ep-CAM in Y79 cells after 5 days of AZC treatment (**J**). After day 5, Y79 cells were withdrawn from AZC exposure and subsequently passaged in the complete medium up to 30 days, and Ep-CAM mRNA expression was retained up to 21 days as follows: 210-fold, 132-fold, 56-fold, and 20-fold on days 8, 10, 15, and 20 days, respectively. The error bars represents the standard deviation of triplicate values (**J**).

We previously have observed increased mRNA expression of *Ep-CAM* in RB primary tumors [6] compared to non-neoplastic retina (data not shown). In contrast to increased *Ep-CAM* expression in primary RB tumors, Y79 showed very faint to absent Ep-CAM protein expression by immunohistochemistry and real-time quantitative reverse transcriptase PCR (Q-RT–PCR). Based on earlier studies [16–19] and the presence of CpG island in the promoter region of *Ep-CAM*, we subjected the cell line to a demethylating agent to find out whether low expression of *Ep-CAM* is due to promoter methylation. In an attempt to re-express *Ep-CAM*, we exposed Y79 to AZC, as described in the methods. The flow cytometry analysis showed 47.73%, 44.2%, 42.96%, and 18.27% of Y79 cells positive for Ep-CAM on days 5, 8, 10, and 15 of AZC treatment, respectively (Figure 2A). On day 5, Y79 cells demonstrated strong membrane positivity for Ep-CAM by immunohistochemistry (Figure 2G). After day 5, Y79 cells were withdrawn from AZC exposure and subsequently passaged in the complete medium up to 30 days, and *Ep-CAM* mRNA expression was retained up to 21 days as follows: 210 fold, 132 fold, 56 fold, and 20-fold on days 8, 10, 15, and 20, respectively (Figure 2J). Thus, AZC treatment restored adequate *Ep-CAM* expression, which was sufficient to perform siRNA experiments in Y79 cells.

### EpCAM siRNA treatment decreases proliferation of Y79 cell line

**Figure 3:**
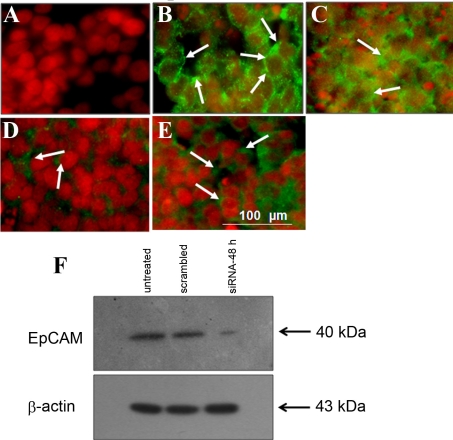
Effect of small interfering RNA on the expression of epithelial cell adhesion molecule in retinoblastoma Y79 cells in vitro. Immunofluorescence picture shows the negative staining without Epithelial cell adhesion molecule (Ep-CAM) antibody in Y79 cells representing as negative control (**A**); Immunofluorescence shows strong positivity (3+; white arrows) for Ep-CAM on day 8 post azacytidine treatment (**B**); After 24 h of short interfering (si)RNA treatment, Y79 cells showed moderate staining (2+; arrows) of Ep-CAM (**C**). After 48 h of siRNA treatment, Y79 cells showed dull staining (1+; arrows) of Ep-CAM (**D**). After 72 h of siRNA treatment, Y79 cells gain Ep-CAM expression (low to moderate intensity). **E**: western blot analysis demonstrates strong expression of Ep-CAM in Y79 cells that are si-RNA untreated (lane 1), strong expression in Y79 cells treated with scrambled siRNA (lane 2) and markedly reduced Ep-CAM expression in Y79 cells treated with Ep-CAM-siRNA (**F**; lane 3).

**Figure 4:**
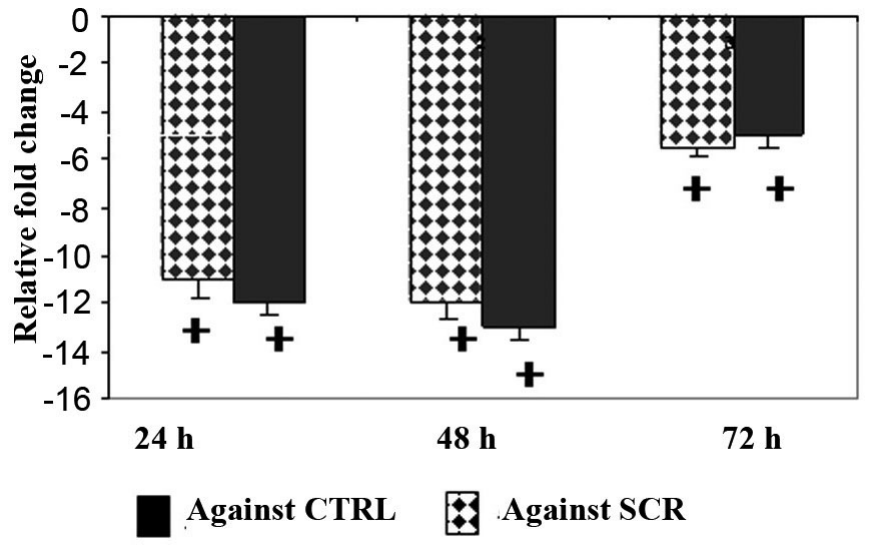
Epithelial cell adhesion molecule- short interfering RNA treatment decreases the mRNA expression of epithelial cell adhesion molecule in Y79 cells. The mRNA levels of Epithelial cell adhesion molecule (Ep-CAM) in Y79 cells treated with Ep-CAM- short interfering (si)RNA was decreased by 11-fold (p<0.001), 12-fold (p<0.001) and 5.5 fold (p<0.01) at 24, 48, and 72 h respectively when compared with Ep-CAM mRNA levels in Y79 cells treated with scrambled (SCR) siRNA (dotted bars). The mRNA levels of Ep-CAM in Y79 cells treated with Ep-CAM-siRNA was decreased by 11-fold (p<0.001), 12-fold (p<0.001) and fivefold (p<0.01) at 24, 48, and 72 h, respectively, when compared with Ep-CAM mRNA levels in control (CTRL) Y79 cells (solid bars). The error bars represents the standard deviation of triplicate values and the statistical analysis was performed using independent *t*-test.

**Figure 5:**
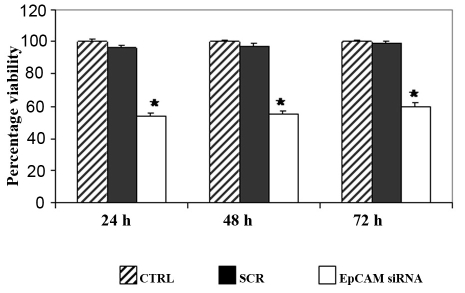
epithelial cell adhesion molecule short interfering RNA treatment decreases the cell proliferation of Y79 cells. The Y79 cells were treated with epithelial cell adhesion molecule epithelial cell adhesion molecule epithelial cell adhesion molecule (Ep-CAM) short interfering (si)RNA and the cell proliferation was assessed at 24, 48, and 72 h using 3-(4,5-dimethylthiazol-2-yl)-2,5-diphenyltetrazolium bromide (MTT) assay. The viability of Y79 cells was 54% (p<0.01), 55% p<0.01) and 60% (p<0.01) at 24, 48, and 72 h of siRNA treatment respectively when compared to Y79 cells that were not treated with siRNA. The error bars represents the standard deviation of triplicate values and the statistical analysis was performed using independent *t*-test.

To determine whether *Ep-CAM* is a potential target for RB gene therapy, the Y79 cell line was treated with *Ep-CAM*-specific siRNA as well as with scrambled siRNA. The scrambled siRNA served as a control for nonspecific effects of siRNA treatment on cell growth. Ep-CAM protein expression, mRNA levels, and cell viability were determined at 24, 48, and 72 h using immunofluorescence (Figure 3A-E), real-time RT–PCR (Figure 4), and MTT assay (Figure 5), respectively. The data show that *Ep-CAM* siRNA treatment results in 12–13 fold decrease in *Ep-CAM* gene expression in the Y79 cell line compared with untreated and scrambled siRNA-treated Y79 cells at 48 h. Scrambled siRNA treatment had no effect on *Ep-CAM* mRNA levels, thus supporting the specificity of the *Ep-CAM* siRNA. Similarly, western blot analysis showed marked decrease in Ep-CAM expression (3.5 fold downregulated when compared to untreated) at 48 h of *Ep-CAM* siRNA treatment, whereas no change was shown when treated with scrambled siRNA (Figure 3F). The *Ep-CAM* siRNA treatment effect was maximal at 48 h and attenuated by 72 h (consistent with the known transient effects of siRNA in mammalian cells). *Ep-CAM* siRNA treatment resulted in a significant decrease in cell proliferation by more than 50% at all time points.

### cDNA microarray

**Figure 6:**
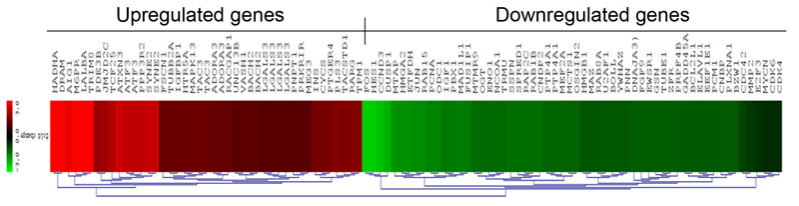
Epithelial cell adhesion molecule short interfering RNA treatment leads to changes in gene expression profile in Y79 cells. The heat map represents the expression profile of 100 genes differentially modified in response to knockdown of epithelial cell adhesion molecule (Ep-CAM) in Y79 cells compared to untreated cells. The horizontal lines represent the relative fold change in the expression of individual genes modified by the Ep-CAM-short interfering (si)RNA. Red and green indicate increased and decreased expression, respectively, relative to nonsilencing control siRNA.

*Ep-CAM* expression was decreased by 12–13 fold, which was minimum at 48 h after siRNA treatment; hence at this time point cells were harvested for microarray analysis. The genome-wide expression profile of *Ep-CAM* siRNA-treated AZC-Y79 cells against siRNA-untreated AZC-Y79 was analyzed to investigate the molecules involved in Ep-CAM signaling. In Y79 cells, anti-Ep-CAM siRNA treatment modulated the expression of 670 gene transcripts (probe sets), 465 (69.4%) of which were increased and 205 (30.5%) were decreased. Only genes with p≤0.05 and log ratio of at least 2.0 for upregulation and log ratio of 0.5 for downregulation, keeping a median log ratio of 1 in both biologic replicates, were considered for expression analysis. The important upregulated and downregulated genes post *Ep-CAM* knockdown (Figure 6) are provided in Table 1 and Table 2, respectively. The data discussed in this publication have been deposited in NCBI's Gene Expression Omnibus [20] and are accessible through GEO Series accession number GSE16991.

**Table 1:** Upregulated genes identified by microarray post EpCAM knock-down in Y79

**Gene classification/description**	**Gene symbol**	**Fold change**	**GenBank accession**	**Chromosome location**
**Cell cycle and cell differentiation**				
Homo sapiens lectin, galactoside-binding, soluble, 3 (LGALS3), transcript variant 1, mRNA	LGALS3	1.036256	[NM_002306]	14q22.3
Homo sapiens 5-hydroxytryptamine (serotonin) receptor 5A (HTR5A), mRNA	HTR5A	1.283948	[NM_024012]	7q36.2
Homo sapiens insulin (INS), mRNA	INS	1.370663	[NM_000207]	11p15.5
Homo sapiens tachykinin 3 (neuromedin K, neurokinin beta) (TAC3), transcript variant 1, mRNA	TAC3	1.281899	[NM_001006667]	12q13.3
Homo sapiens activating transcription factor 3 (ATF3), transcript variant 2, mRNA	ATF3	2.262722	[NM_004024]	1q32.3
**Apoptosis**				
Homo sapiens lactalbumin, alpha- (LALBA), mRNA	LALBA	3.158096	[NM_002289]	12q13.2
Homo sapiens retinoic acid receptor, gamma (RARG), transcript variant 1, mRNA	RARG	1.541636	[NM_000966]	12q13.13
Homo sapiens ataxin 3 (ATXN3), transcript variant 1, mRNA	ATXN3	2.464725	[NM_004993]	14q32.12
Homo sapiens spectrin repeat containing, nuclear envelope 2 (SYNE2), transcript variant 2, mRNA	SYNE2	2.345608	[NM_182910]	14q23.2
Homo sapiens mitogen-activated protein kinase 13 (MAPK13), mRNA	MAPK13	1.283948	[NM_002754]	6p21.31
Homo sapiens tubulin, beta 2A (TUBB2A), mRNA	TUBB2A	1.269632	[NM_001069]	6p25.2
Homo sapiens plastin 3 (T isoform) (PLS3), mRNA	PLS3	1.596833	[NM_005032]	Xq23
Homo sapiens unc-13 homolog B (C. elegans) (UNC13B), mRNA	UNC13B	1.084631	[NM_006377]	9p13.3
Homo sapiens BTB and CNC homology 1, basic leucine zipper transcription factor 2 (BACH2), mRNA	BACH2	1.111043	[NM_021813]	6q15
Homo sapiens activating transcription factor 3 (ATF3), transcript variant 2, mRNA	ATF3	2.262722	[NM_004024]	1q32.3
Homo sapiens insulin-like growth factor binding protein 1 (IGFBP1), transcript variant 1, mRNA	IGFBP1	1.261051	[NM_000596]	7p13
Homo sapiens protein phosphatase 3 (formerly 2B), regulatory subunit B, beta isoform (PPP3R2), mRNA	PPP3R2	2.201001	[NM_147180]	9q31.1
Homo sapiens androgen-induced 1 (AIG1), mRNA	AIG1	2.769478	[NM_016108]	6q24.2
Homo sapiens mannose-6-phosphate receptor (cation dependent) (M6PR), mRNA	M6PR	2.997706	[NM_002355]	12p13.31
Homo sapiens hydroxyacyl-CoA dehydrogenase/3-ketoacyl-CoA thiolase/enoyl-CoA hydratase (trifunctional protein), alpha subunit (HADHA), nuclear gene encoding mitochondrial protein, mRNA	HADHA	4.116386	[NM_000182]	2p23.3
Homo sapiens transcription factor 25 (basic helix–loop–helix)	TCF25	2.112025	[NM_014972]	16q24.3
Homo sapiens protein-kinase, interferon-inducible double stranded RNA dependent inhibitor, repressor of (P58 repressor) (PRKRIR), mRNA	PRKRIR	1.065873	[NM_004705]	8q21.12
Homo sapiens cytochrome c, somatic (CYCS), nuclear gene encoding mitochondrial protein, mRNA	CYCS	1.40148	[NM_018947]	7p15.2
Homo sapiens damage-regulated autophagy modulator (DRAM), mRNA	DRAM	4.908309	[NM_018370]	12q23.2
Homo sapiens mRNA; cDNA DKFZp762K1914 (from clone DKFZp762K1914).	PNPT1	1.054391	[CR749867]	2p16.1
**Anti-Apoptosis**				
Homo sapiens phosphodiesterase 3B, cGMP-inhibited (PDE3B), mRNA	PDE3B	1.798894	[NM_000922]	11p15.2
Homo sapiens lectin, galactoside-binding, soluble, 3 (LGALS3), transcript variant 1, mRNA	LGALS3	1.036256	[NM_002306]	14q22.3
Homo sapiens adenosine A3 receptor (ADORA3), transcript variant 2, mRNA	ADORA3	1.177361	[NM_000677]	1p13.2
Homo sapiens prostaglandin E receptor 4 (subtype EP4) (PTGER4), mRNA	PTGER4	1.424538	[NM_000958]	5p13.1
**Angiogenesis**				
Homo sapiens fascin homolog 1, actin-bundling protein (Strongylocentrotus purpuratus) (FSCN1), mRNA	FSCN1	1.247566	[NM_003088]	7p22.1
**Anti-angiogenesis**				
Homo sapiens lectin, galactoside-binding, soluble, 3 (LGALS3), transcript variant 1, mRNA	LGALS3	1.036256	[NM_002306]	14q22.3
Homo sapiens vasohibin 1 (VASH1), mRNA	VASH1	1.085812	[NM_014909]	14q24.3
**Proliferation**				
Homo sapiens jumonji domain containing 2C (JMJD2C), mRNA [NM_015061]	JMJD2C	1.889542	[NM_015061]	9p24.1
Homo sapiens adenosine A3 receptor (ADORA3), transcript variant 2, mRNA	ADORA3	1.177361	[NM_000677]	1p13.2
Homo sapiens tachykinin 3 (neuromedin K, neurokinin beta) (TAC3), transcript variant 1, mRNA	TAC3	1.281899	[NM_013251]	12q13.3
**Anti-proliferation**				
Homo sapiens spectrin repeat containing, nuclear envelope 2 (SYNE2), transcript variant 2, mRNA	SYNE2	2.345608	[NM_182910]	14q23.2
Homo sapiens lectin, galactoside-binding, soluble, 3 (LGALS3), transcript variant 1, mRNA	LGALS3	1.036256	[NM_002306]	14q22.3
Homo sapiens BTB and CNC homology 1, basic leucine zipper transcription factor 2 (BACH2), mRNA	BACH2	1.111043	[NM_021813]	6q15
Homo sapiens tripartite motif-containing 8 (TRIM8), mRNA	TRIM8	3.145901	[NM_030912]	10q24.32
Homo sapiens Rac GTPase activating protein 1 (RACGAP1), mRNA	RACGAP1	1.167279	[NM_013277]	12q13.13
Homo sapiens cDNA FLJ41884 fis, clone OCBBF2023162.	MEG3	1.066443	[AK123878]	14q32.2
**Tumor suppressor**				
Homo sapiens tropomyosin 1 (alpha) (TPM1), transcript variant 6, mRNA	TPM1	1.539701	[NM_001018008]	15q22.2

**Table 2:** Down-regulated genes identified by microarray post EpCAM Knock-down in Y79

**Gene classification/description**	**Gene symbol**	**Fold change**	**GenBank accession**	**Chromosome location**
**Proliferation**				
Homo sapiens fibroblast growth factor 9 (glia-activating factor)	FGF9	−1.09836	[NM_002010]	13q12.11
Homo sapiens v-fos FBJ murine osteosarcoma viral oncogene homolog (FOS)	FOS	−2.42289	[NM_005252]	14q24.3
Homo sapiens jun oncogene (JUN)	JUN	−1.65468	[NM_002228]	1p32.1
Homo sapiens E2F transcription factor 3 (E2F3), mRNA	E2F3	−0.57954	[NM_001949]	6p22.3
Homo sapiens proliferating cell nuclear antigen (PCNA), transcript variant 1, mRNA	PCNA	−1.53558	[NM_002592]	20p12.3
Homo sapiens high mobility group AT-hook 2 (HMGA2), transcript variant 1, mRNA	HMGA2	−1.70144	[NM_003483]	12q14.3
Homo sapiens hairy and enhancer of split 1, (Drosophila) (HES1), mRNA	HES1	−2.41369	[NM_005524]	3q29
Homo sapiens cyclin D3 (CCND3), mRNA	CCND3	−2.27265	[NM_001760]	6p21.1
Homo sapiens growth arrest and DNA-damage-inducible, alpha (GADD45A), mRNA	GADD45A	−1.0626	[NM_001924]	1p31.3
Homo sapiens ELAV (embryonic lethal, abnormal vision, Drosophila)-like 1 (Hu antigen R) (ELAVL1), mRNA	ELAVL1	−1.04274	[NM_001419]	19p13.2
Homo sapiens nuclear receptor coactivator 1 (NCOA1), transcript variant 3, mRNA	NCOA1	−1.24715	[NM_147233]	2p23.3
Homo sapiens electron-transferring-flavoprotein dehydrogenase (ETFDH), nuclear gene encoding mitochondrial protein, mRNA	ETFDH	−1.69508	[NM_004453]	4q32.1
Homo sapiens myotubularin related protein 9 (MTMR9), mRNA	MTMR9	−1.26689	[NM_015458]	8p23.1
Homo sapiens protein tyrosine phosphatase type IVA, member 1 (PTP4A1), mRNA	PTP4A1	−1.18325	[NM_003463]	6q12
Homo sapiens CCHC-type zinc finger, nucleic acid binding protein (CNBP), mRNA	CNBP	−1.07756	[NM_003418]	3q21.3
Homo sapiens enolase 1, (alpha) (ENO1), mRNA	ENO1	1.24292	[NM_001428]	1p36.23
Homo sapiens insulin-like growth factor 1 (somatomedin C) (IGF1), mRNA	IGF1	−1.41077	[NM_000618]	12q23.2
Homo sapiens Ewing sarcoma breakpoint region 1, mRNA (cDNA clone MGC:8607 IMAGE:2961279)	EWSR1	−1.10194	[BC000527]	22q12.2
Homo sapiens sprouty-related, EVH1 domain containing 1 (SPRED1), mRNA	SPRED1	−1.28822	[NM_152594]	15q14
**Cell invasion**				
Homo sapiens cell division cycle 42 (GTP binding protein, 25 kDa) (CDC42), transcript variant 2, mRNA	CDC42	−0.79444	[NM_044472]	1p36.12
Homo sapiens matrix metallopeptidase 2 (gelatinase A, 72 kDa gelatinase, 72 kDa type IV collagenase) (MMP2), mRNA	MMP2	−0.67629	[NM_004530]	16q12.2
Homo sapiens plexin A1 (PLXNA1), mRNA	PLXNA1	−1.07618	[NM_032242]	3q21.3
Homo sapiens protein tyrosine phosphatase type IVA, member 1 (PTP4A1), mRNA	PTP4A1	−1.18325	[NM_003463]	6q12
Homo sapiens MADS box transcription enhancer factor 2, polypeptide A (myocyte enhancer factor 2A) (MEF2A), mRNA	MEF2A	−1.17285	[NM_005587]	15q26.3
Homo sapiens methylthioadenosine phosphorylase (MTAP), mRNA	MTAP	−1.75238	[NM_002451]	9p21.3
Homo sapiens tyrosine 3-monooxygenase/tryptophan 5-monooxygenase activation protein, zeta polypeptide (YWHAZ), transcript variant 2, mRNA	YWHAZ	−1.02872	[NM_145690]	8q22.3
**Angiogenesis**				
Homo sapiens dual specificity phosphatase 1 (DUSP1), mRNA	DUSP1	−2.09785	[NM_004417]	5q35.1
Homo sapiens malignant T cell amplified sequence 1 (MCTS1), mRNA	MCTS1	−1.16085	[NM_014060]	Xq24
**Oncogene**				
Homo sapiens RAP2C, member of RAS oncogene family (RAP2C), mRNA	RAP2C	−1.29212	[NM_021183]	Xq26.2
Homo sapiens RAB8A, member RAS oncogene family (RAB8A), mRNA	RAB8A	−1.02394	[NM_005370]	19p13.12
Homo sapiens sarcospan (Kras oncogene-associated gene) (SSPN), mRNA	SSPN	−1.31047	[NM_005086]	12p12.1
Homo sapiens RAB1B, member RAS oncogene family (RAB1B), mRNA	RAB1B	−1.29331	[NM_030981]	11q13.1
Homo sapiens RAB15, member RAS onocogene family (RAB15), mRNA	RAB15	−1.64564	[NM_198686]	14q23.3
Homo sapiens v-myc myelocytomatosis viral related oncogene, neuroblastoma derived (avian) (MYCN), mRNA	MYCN	−0.50087	[NM_005378]	2p24.3
**Antiapoptotic**				
Homo sapiens DnaJ (Hsp40) homolog, subfamily A, member 3 (DNAJA3), mRNA	DNAJA3)	−1.11925	[NM_005147]	16p13.3
Homo sapiens ornithine decarboxylase 1 (ODC1), mRNA	ODC1	−1.56073	[NM_002539]	2p25.1
Homo sapiens gelsolin (amyloidosis, Finnish type) (GSN), transcript variant 2, mRNA	GSN	−1.1058	[NM_198252]	9q33.2
Homo sapiens BCL2-like 1 (BCL2L1), nuclear gene encoding mitochondrial protein, transcript variant 2, mRNA	BCL2L1	−1.06389	[NM_001191]	20q11.21
Homo sapiens high-mobility groupbox 1 (HMGB1), mRNA	HMGB1	−1.00917	[NM_002128]	13q12.3
**Cell cycle and differentiation**				
Homo sapiens bol, boule-like (Drosophila) (BOLL), transcript variant 2, mRNA	BOLL	−1.03287	[NM_033030]	2q33.1
Homo sapiens tubulin, epsilon 1 (TUBE1), mRNA	TUBE1	−1.10675	[NM_016262]	6q21
Homo sapiens eukaryotic translation elongation factor 1 epsilon 1 (EEF1E1), mRNA [NM_004280]	EEF1E1	−1.04824	[NM_004280]	6p24.3
Homo sapiens MAD1 mitotic arrest deficient-like 1 (yeast) (MAD1L1), transcript variant 1, mRNA	MAD1L1	−1.48937	[NM_003550]	7p22.3
Homo sapiens MYC-associated zinc finger protein (purine-binding transcription factor) (MAZ), transcript variant 1, mRNA	MAZ	−1.00923	[NM_002383]	16p11.2
Homo sapiens O-linked N-acetylglucosamine (GlcNAc) transferase (UDP-N-acetylglucosamine:polypeptide-N-acetylglucosaminyl transferase) (OGT), transcript variant 1, mRNA	OGT	−1.2658	[NM_181672]	Xq13.1
Homo sapiens U2 small nuclear RNA auxiliary factor 1 (U2AF1), transcript variant a, mRNA	U2AF1	−1.02281	[NM_006758]	21q22.3
Homo sapiens tRNA 5-methylaminomethyl-2-thiouridylate methyltransferase (TRMU), nuclear gene encoding mitochondrial protein, transcript variant 1, mRNA	TRMU	−1.24944	[NM_018006]	22q13.31
Homo sapiens CNDP dipeptidase 2 (metallopeptidase M20 family) (CNDP2), mRNA	CNDP2	−1.29951	[NM_018235]	18q22.3
Homo sapiens pinin, desmosome associated protein (PNN), mRNA	PNN	−1.02816	[NM_002687]	14q21.1
Homo sapiens pericentriolar material 1 (PCM1), mRNA	PCM1	−1.05392	[NM_006197]	8p22
Pre-B-cell leukemia transcription factor 1 (Homeobox protein PBX1) (Homeobox protein PRL).	PBX1	−1.42582	[NM_002585]	1q23.3
Homo sapiens oxidative stress induced growth inhibitor family member 2 (OSGIN2), mRNA	OSGIN2	−1.15358	[NM_004337]	8q21.3
Homo sapiens zinc finger RNA binding protein (ZFR), mRNA	ZFR	−1.10655	[NM_016107]	5p13.3
Homo sapiens PRP4 pre-mRNA processing factor 4 homolog B (yeast) (PRPF4B), mRNA	PRPF4B	−1.10445	[NM_003913]	6p25.2
Homo sapiens FUS interacting protein (serine/arginine-rich) 1 (FUSIP1), transcript variant 2, mRNA	FUSIP1	−1.45444	[NM_054016]	1p36.11
Homo sapiens basic leucine zipper and W2 domains 1 (BZW1), mRNA	BZW1	−1.07595	[NM_014670]	2q33.1
Homo sapiens cyclin-dependent kinase 6 (CDK6), mRNA	CDK6	−0.5	[NM_001259]	7q21.2
Homo sapiens cyclin-dependent kinase 4 (CDK4), mRNA	CDK4	−0.513	[NM_000075]	12q14.1

### Upregulated genes post epithelial cell adhesion molecule knockdown

In cells with inhibited *Ep-CAM* expression, upregulated categories represented gene products that were involved in apoptosis, such as lactalbumin, alpha (*LALBA*)*,* retinoic acid receptor, gamma (*RARG*), ataxin 3 (*ATXN3*)*,* mitogen-activated protein kinase 13 (*MAPK13*), tubulin, beta 2A (*TUBB2A*), activating transcription factor 3 (*ATF3*)*,* insulin-like growth factor binding protein 1 (*IGFBP1*)*,* Homo sapiens damage-regulated autophagy modulator (*DRAM*), and cytochrome c, somatic (*CYCS*); anti-angiogenic genes, such as lectin, galactoside-binding, soluble, 3 (*LGALS3*)*,* and vasohibin 1 (*VASH1*); cell cycle and differentiation genes, such as *LGALS3,* 5-hydroxytryptamine (serotonin) receptor 5A (*HTR5A*)*, INS*, tachykinin 3 [neuromedin K, neurokinin beta] (*TAC3*)*,* and *ATF3*; anti-proliferation genes, such as spectrin repeat containing, nuclear envelope 2 (*SYNE2*)*,* BTB and CNC homology 1, basic leucine zipper transcription factor 2 (*BACH2*)*,* tripartite motif-containing 8 (*TRIM8*)*,* Rac GTPase activating protein 1 (*RACGAP1*)*,* and *MEG3*; and tumor suppressor gene tropomyosin 1 alpha (*TPM1*).

### Downregulated genes post epithelial cell adhesion molecule silencing

Downregulated categories represented gene products that were involved in cell cycle and cell division genes, such as MYC-associated zinc finger protein [purine-binding transcription factor] (*MAZ*)*,* tubulin, epsilon 1 (*TUBE1*)*, MAD1,* sprouty-related, EVH1 domain containing 1 (*SPRED1*), U2 small nuclear RNA auxiliary factor 1 (*U2AF1*)*, CNDP,* and stress induced growth inhibitor family member 2 (*SGIN2*); cell migration genes, such as *MMP2,* cell division cycle 42 [GTP binding protein, 25 kDa] (*cdc42*)*,* and MADS box transcription enhancer factor 2, polypeptide A [myocyte enhancer factor 2A] (*MEF2A)*; anti-apoptotic genes, such as DnaJ (Hsp40) homolog, subfamily A, member 3 (*DNAJA3*)*, *ornithine decarboxylase 1 (*ODC1*)*,* gelsolin [amyloidosis, Finnish type] (*GSN*), BCL2-like 1 (*BCL21*), and high-mobility groupbox 1 (*HMGB1*); proliferation genes, such as *FGF9,* *Homo sapiens* v-fos FBJ murine osteosarcoma viral oncogene homolog (*FOS*), *Homo sapiens* jun oncogene (*JUN*)*, PCNA,* *Homo sapiens* E2F transcription factor 3 (*E2F3*), malignant T cell amplified sequence 1 (*MCTS1*)*,* hairy and enhancer of split 1, [*Drosophila*] (*HES1*), cyclin D3 (*CCND3*), growth arrest and DNA-damage-inducible, alpha (*GADD45A*)*,* embryonic lethal, abnormal vision, Drosophila-like 1 [Hu antigen R] (*ELAVL1*), nuclear receptor coactivator 1 (*NCOA1*)*,* enolase 1, [alpha] (*ENO1*), and insulin-like growth factor 1 [somatomedin C] (*IGF1*); and oncogenes, such as *FOS,* member of RAS oncogene family (*RAP2C*)*,* member RAS oncogene family (*RAB8A*)*, JUN,* sarcospan [Kras oncogene-associated gene] (*SSPN*)*, RAB1B, RAB15,* and v-myc myelocytomatosis viral related oncogene, neuroblastoma derived [avian] (*MCYN*).

### Deregulated pathways post epithelial cell adhesion molecule silencing

Upon *Ep-CAM* inhibition, MAP kinase pathway was deregulated in Y79 cells. The genes involved in MAP kinase pathway were *FOS, JUN, FGF9,* and *GADD45A*. On the other hand, P53 pathway molecules were upregulated upon *Ep-CAM* inhibition. The molecules involved in P53 pathway were *RRM2, CYCS*, and *DRAM*.

### Functional grouping of differentially expressed genes

**Figure 7:**
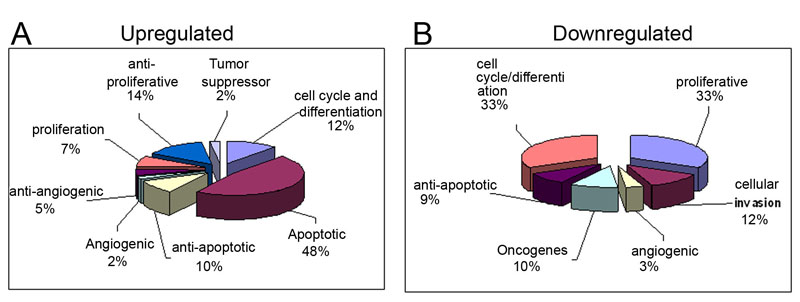
Functional grouping of upregulated genes. (**A**) and downregulated genes (**B**) in Y79 cells treated with epithelial cell adhesion molecule short interfering (si)RNA. All the distinct gene identifications were examined for their known biologic function according to gene ontology convention and grouped in the respective functional category. The proportion of each functional category in the total number of selected identified genes (taken as 100%) is shown.

All the distinct gene identifications were examined for their known biologic function according to gene ontology convention and grouped in the respective functional category. The proportion of each functional category in the total number of selected identified genes (taken as 100%) is shown in Figure 7. Among the upregulated genes, (Figure 7A) the majority of genes belong to the apoptosis functional category (48%). Hence, it is interesting to speculate that Ep-CAM inhibition may promote apoptosis in Y79 cells. Other genes identified belong to proliferation, anti-proliferation, angiogenesis, anti-angiogenesis, anti-apoptotic, and tumor suppressor genes. Among the downregulated genes, (Figure 7B) one-third of the total genes belong to the proliferation functional category (33%) and cell cycle and differentiation (33%). Other downregulated genes belong to cellular invasion, anti-apoptosis, oncogenes, and angiogenic genes.

### Real-time quantitative reverse transcriptase-PCR to confirm microarray data

**Figure 8:**
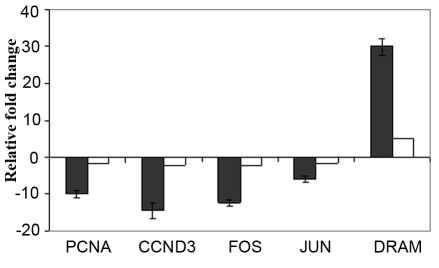
The mRNA expression of selected genes from microarray data was confirmed using real time quantitative reverse transcriptase PCR (Q-RT–PCR). The black bars represent the mRNA levels quantified by Q-RT–PCR and the white bars represent the fold expression of genes studied by microarray. The Q-RT–PCR results are consistent with microarray results. The error bars represent the standard deviation of triplicate values. Abbreviations: PCNA represents proliferating cell nuclear antigen, CCDN3 represents cyclin D3, FOS represents v-fos FBJ murine osteosarcoma viral oncogene homolog, JUN represents jun oncogene, and DRAM represents damage-regulated autophagy modulator.

Five genes (*PCNA, CCND3, FOS, JUN*, and *DRAM*) from microarray data have been confirmed by real-time Q-RT–PCR. The results are consistent with the microarray data. The gene expression of all five genes studied was higher when measured using Q-RT–PCR as compared to microarray analysis, reflecting the better dynamic range of Q-RT–PCR. The relative mRNA expression of *DRAM* was significantly increased, and the relative mRNA expression of *PCNA, CCND3, FOS*, and *JUN* were significantly downregulated in siRNA-treated Y79 cells compared to untreated Y79 cells (Figure 8).

## Discussion

To investigate the functional relevance of Ep-CAM in RB, we proposed to transiently silence *Ep-CAM* expression using siRNA and study its effect on whole gene expression profiling. We chose the Y79 cell line for in vitro studies for two reasons. One reason is that Y79 cells form tumors in the murine model that closely resemble the naturally occurring tumor in anatomic sites without disrupting the choroid or sclera or involving the anterior chamber [21]. The second reason is that as RB tumors from Indian RB patients are advanced tumors, presenting with choroidal and optic nerve invasion [22], the Y79 cell line is appropriate for in vitro studies and the data can be translated to the actual tumor state.

Our study shows that both mRNA and protein levels of Ep-CAM are very low to absent in Y79 cells. There is substantial evidence that DNA methylation plays an important role in silencing specific genes during development and cell differentiation [23,24]. Earlier studies have shown that *Ep-CAM* is regulated by the most common epigenetic mechanism—hypermethylation in oral squamous cell carcinoma and breast carcinomas [16–19]. Epigenetic silencing of a gene can be reversed by drugs, such as AZC, which acts through incorporation of the modified nucleotide in the place of cytosine into the newly synthesized strand during DNA replication. The modified nucleotides form a covalent complex with the active sites of the DNA methyltransferase, depleting methyltransferase activity, resulting in generalized demethylation [25]. In this context, we restored *Ep-CAM* expression in Y79 cells by subjecting the cells to demethylation (AZC treatment) in vitro for 5 days. However, these drugs have effects other than inducing demethylation. The amount of methylation and the response obviously can vary for the same gene from one cell line to another and between different genes in the same cell line. One disadvantage of a drug-based reactivation approach is that, unlike CpG island array screening, use is essentially restricted to cell cultures and is not amenable to examination of primary tumors.

In the present study, AZC treatment restored adequate *Ep-CAM* expression, which was sufficient to perform siRNA experiments in Y79 cells. Silencing of the *Ep-CAM* gene significantly decreased the proliferative capacity of RB Y79 cells. Expression microarray analysis of *Ep-CAM* silencing revealed several genes related to cell proliferation, apoptosis, anti-apoptosis, cell division and differentiation, angiogenesis, anti-angiogenesis, and oncogenes.

Cells expressing *Ep-CAM* proliferate more rapidly, grow in an anchorage-independent manner, and have a reduced requirement for growth factors. Consistent with the growth-promoting role of Ep-CAM, knockdown of endogenous *Ep-CAM* in tumor cells decreases cell proliferation and migration [7,26]. The role of *Ep-CAM* in proliferation and its association with cancer is poorly explained by proposed cell adhesion functions. Recently, Maetzel et al. [27] showed that regulated intramembrane proteolysis activates Ep-CAM as a mitogenic signal transducer in vitro and in vivo. The cleaved intracellular domain Ep-CAM intracellular domain (EpICD) associates with four and a half LIM domain (FHL2), β-catenin, and lymphoid enhancer binding factor 1 (Lef-1) to form a nuclear complex that contacts DNA at Lef-1 consensus sites, induces gene transcription, and is oncogenic in immunodeficient mice. Hence, this mechanism of nuclear signaling of Ep-CAM explains how it functions in cell proliferation.

Our study showed significant downregulation of PCNA post-*Ep-CAM* silencing. A high PCNA index has been noted in RB in a recent study by Dimaras et al. [28]. In gastric cancer, the PCNA index of gastric cancer tissues with high *Ep-CAM* expression was higher than gastric cancer tissues with low *Ep-CAM* expression, indicating that *Ep-CAM* might be associated with the proliferative activity of cancer cells [29]. We also showed that *FOS* and *JUN*, which are involved in cellular proliferation, were downregulated post-*Ep-CAM* silencing.

Genes related to apoptosis, such as damage-regulated autophagy modulator DRAM, a critical effector of p53-induced autophagy [30], and cytochrome *c* CYCS, a central molecule in apoptotic signaling [31], were significantly upregulated. Recently, a study on mammary tissues in transgenic mice showed that *Ep-CAM* overexpression could induce K_i_-67 upregulation and reduce apoptosis of cells, accompanied by upregulation of *bcl-2* expression, which was the first report about the relation between Ep-CAM and apoptosis [32]. Our observations indicate that Ep-CAM inhibition on cellular proliferation is by cell cycle arrest accompanied by apoptosis. However, further functional studies need to be conducted to prove these effects in RB.

These changes are coupled with downregulation of important cell cycle-related transcription factors, *E2F3* and *CCND3*. E2Fs are transcription factors best known for their involvement in the timely regulation of gene expression required for cell cycle progression. *E2F3* overexpression has been previously demonstrated in RB tumors by Gallie et al. [33]. Cyclins are periodic regulatory proteins governing cell cycle transit from G1 phase into S phase. Overexpression of cyclins could lead to abnormal cellular proliferation, which underlies the process of tumorigenesis [34].

Interestingly, our study showed the downregulation of *MYCN* post-*Ep-CAM* silencing. *MYCN* is a well studied oncogene known to be amplified in neuroblastoma [35] and in a subset of RB tumors [36,37]. Previously it was demonstrated that Ep-CAM has a direct impact on the cell cycle and proliferation and the ability to rapidly upregulate the proto-oncogene *c-myc* and *cyclin A/E* [21]. However, further studies are needed to establish the *MYCN* relation with *Ep-CAM* signaling in RB.

In addition to proliferation and apoptotic genes, we showed that genes related to tumor invasion, metastasis, and angiogenesis, such as MMPs and *cdc42* are significantly downregulated post *Ep-CAM* silencing. Previously we demonstrated high MMP [38] and cdc42 [39] expression in RB primary tumors. The downregulation of MMPs in the present study supports the hypothesis that *Ep-CAM* inhibition could reduce tumor cell invasion, as demonstrated in an earlier study [7]. *Ep-CAM* inhibition led to downregulation of the MAP kinase pathway in Y79 cells. The genes involved in the MAP kinase pathway are *FOS*, *JUN*, *FGF9*, and *GADD45A*. On the other hand, P53 pathway molecules were upregulated upon *Ep-CAM* inhibition. The molecules involved in the P53 pathway are RRM2, CYCS, and DRAM.

In conclusion, we showed for the first time that *Ep-CAM* silencing in RB in vitro leads to a decrease in Y79 cell proliferation and deregulation of several genes related to cell survival/proliferation, DNA replication/transcription, apoptosis, and angiogenesis. Targeting *Ep-CAM* for molecular intervention appears to be an attractive strategy. First, *Ep-CAM* is substantially overexpressed in primary RB tumor samples [6]. Furthermore, because *Ep-CAM* overexpression is associated with proliferation and neoplastic transformation [40,41], silencing of *Ep-CAM* gene expression is likely to dramatically alter the phenotype of cancer cells without significantly influencing normal or nonproliferating cells. Future studies targeting *Ep-CAM* gene expression in vivo will help to delineate the mechanisms associated with *Ep-CAM* gene function in neoplastic transformation and define the potential for *Ep-CAM*-based molecular intervention in RB patients.

## References

[1] Suzuki S Kaneko A Management of intraocular retinoblastoma and ocular prognosis. Int J Clin Oncol 2004 9 1 6 15162819 10.1007/s10147-003-0366-0

[2] Kremens B Wieland R Reinhard H Neubert D Beck JD Klingebiel T Bornfeld N Havers W High-dose chemotherapy with autologous stem cell rescue in children with retinoblastoma. Bone Marrow Transplant 2003 31 281 4 12621463 10.1038/sj.bmt.1703832

[3] Grabowski EF Abramson DH Intraocular and extraocular retinoblastoma. Hematol Oncol Clin North Am 1987 1 721 35 3323180

[4] Doz F Khelfaoui F Mosseri V Validire P Quintana E Michon J Desjardins L Schlienger P Neuenschwander S Vielh P Putterman M Dufier J-L Jean-Michael Zucker The role of chemotherapy in orbital involvement of retinoblastoma. The experience of a single institution with 33 patients. Cancer 1994 74 722 32 8033054 10.1002/1097-0142(19940715)74:2<722::aid-cncr2820740228>3.0.co;2-h

[5] Schvartzman E Chantada G Fandiño A de Dávila MT Raslawski E Manzitti J Results of a stage-based-protocol for the treatment of retinoblastoma. J Clin Oncol 1996 14 1532 6 8622068 10.1200/JCO.1996.14.5.1532

[6] Krishnakumar S Mohan A Mallikarjuna K Venkatesan N Biswas J Shanmugam MP Ren-Heidenreich L Ep-CAM expression in retinoblastoma: a novel molecular target for therapy. Invest Ophthalmol Vis Sci 2004 45 4247 50 15557427 10.1167/iovs.04-0591

[7] Osta WA Chen Y Mikhitarian K Mitas M Salem M Hannun YA Cole DJ Gillanders WE Ep-CAM is overexpressed in breast cancer and is a potential target for breast cancer gene therapy. Cancer Res 2004 64 5818 24 15313925 10.1158/0008-5472.CAN-04-0754

[8] Winter MJ Nagtegaal ID van Krieken JH Litvinov SV The epithelial cell adhesion molecule (Ep-CAM) as a morphoregulatory molecule is a tool in surgical pathology. Am J Pathol 2003 163 2139 48 14633587 10.1016/S0002-9440(10)63570-5 PMC1892395

[9] Koprowski H Steplewski Z Mitchell K Herlyn M Herlyn D Fuhrer P Colorectal carcinoma antigens detected by hybridoma antibodies. Somatic Cell Genet 1979 5 957 72 94699 10.1007/BF01542654

[10] Zorzos J Zizi A Bakiras A Pectasidis D Skarlos DV Zorzos H Elemenoglou J Likourinas M Expression of a cell surface antigen recognized by the monoclonal antibody AUA1 in bladder carcinoma: an immunohistochemical study. Eur Urol 1995 28 251 4 8536781 10.1159/000475060

[11] Braun S Hepp F Kentenich CR Janni W Pantel K Riethmüller G Willgeroth F Sommer HL Monoclonal antibody therapy with edrecolomab in breast cancer patients: monitoring of elimination of disseminated cytokeratin-positive tumor cells in bone marrow. Clin Cancer Res 1999 5 3999 4004 10632331

[12] Seligson DB Pantuck AJ Liu X Huang Y Horvath S Bui MH Han KR Correa AJ Eeva M Tze S Belldegrun AS Figlin RA Epithelial cell adhesion molecule (KSA) expression: pathobiology and its role as an independent predictor of survival in renal cell carcinoma. Clin Cancer Res 2004 10 2659 69 15102668 10.1158/1078-0432.ccr-1132-03

[13] de Boer CJ van Krieken JH Janssen-van Rhijn CM Litvinov SV Expression of Ep-CAM in normal, regenerating, metaplastic, and neoplastic liver. J Pathol 1999 188 201 6 10398165 10.1002/(SICI)1096-9896(199906)188:2<201::AID-PATH339>3.0.CO;2-8

[14] Elbashir SM Harborth J Lendeckel W Yalcin A Weber K Tuschl T Duplexes of 21-nucleotide RNAs mediate RNA interference in cultured mammalian cells. Nature 2001 411 494 8 11373684 10.1038/35078107

[15] Livak KJ Schmittgen TD Analysis of relative gene expression data using real-time quantitative PCR and the 2(-Delta Delta C(T)) Method. Methods 2001 25 402 8 11846609 10.1006/meth.2001.1262

[16] van der Gun BT Wasserkort R Monami A Jeltsch A Raskó T Slaska-Kiss K Cortese R Rots MG de Leij LF Ruiters MH Kiss A Weinhold E McLaughlin PM Persistent downregulation of the pancarcinoma-associated epithelial cell adhesion molecule via active intranuclear methylation. Int J Cancer 2008 123 484 9 18398839 10.1002/ijc.23476

[17] Shiah SG Chang LC Tai KY Lee GH Wu CW Shieh YS The involvement of promoter methylation and DNA methyltransferase-1 in the regulation of Ep-CAM expression in oral squamous cell carcinoma. Oral Oncol 2009 45 e1 8 18485790 10.1016/j.oraloncology.2008.03.003

[18] Spizzo G Gastl G Obrist P Fong D Haun M Grünewald K Parson W Eichmann C Millinger S Fiegl H Margreiter R Amberger A Methylation status of the Ep-CAM promoter region in human breast cancer cell lines and breast cancer tissue. Cancer Lett 2007 246 253 61 16624485 10.1016/j.canlet.2006.03.002

[19] Tai KY Shiah SG Shieh YS Kao YR Chi CY Huang E Lee HS Chang LC Yang PC Wu CW DNA methylation and histone modification regulate silencing of epithelial cell adhesion molecule for tumor invasion and progression. Oncogene 2007 26 3989 97 17213811 10.1038/sj.onc.1210176

[20] Edgar R Domrachev M Lash AE Gene Expression Omnibus: NCBI gene expression and hybridization array data repository Nucleic Acids Res 2002 30 207 10 11752295 10.1093/nar/30.1.207 PMC99122

[21] Chévez-Barrios P Hurwitz MY Louie K Marcus KT Holcombe VN Schafer P Aguilar-Cordova CE Hurwitz RL Metastatic and nonmetastatic models of retinoblastoma. Am J Pathol 2000 157 1405 12 11021842 10.1016/S0002-9440(10)64653-6 PMC1850157

[22] Biswas J Das D Krishnakumar S Shanmugam MP Histopathologic analysis of 232 eyes with retinoblastoma conducted in an Indian tertiary-care ophthalmic center. J Pediatr Ophthalmol Strabismus 2003 40 265 7 14560832 10.3928/0191-3913-20030901-05

[23] Riggs AD X inactivation, differentiation, and DNA methylation. Cytogenet Cell Genet 1975 14 9 25 1093816 10.1159/000130315

[24] Singal R Ginder GD DNA methylation. Blood 1999 93 4059 70 10361102

[25] Jones PA Baylin SB The epigenomics of cancer. Cell 2007 128 683 92 17320506 10.1016/j.cell.2007.01.029 PMC3894624

[26] Münz M Kieu C Mack B Schmitt B Zeidler R Gires O The carcinoma-associated antigen Ep-CAM upregulates c-myc and induces cell proliferation. Oncogene 2004 23 5748 58 15195135 10.1038/sj.onc.1207610

[27] Maetzel D Denzel S Mack B Canis M Went P Benk M Kieu C Papior P Baeuerle PA Munz M Gires O Nuclear signalling by tumour-associated antigen Ep-CAM. Nat Cell Biol 2009 11 162 71 19136966 10.1038/ncb1824

[28] Dimaras H Khetan V Halliday W Orlic M Prigoda NL Piovesan B Marrano P Corson TW Eagle RC Jr Squire JA Gallie BL Loss of RB1 induces non-proliferative retinoma: increasing genomic instability correlates with progression to retinoblastoma. Hum Mol Genet 2008 17 1363 72 18211953 10.1093/hmg/ddn024

[29] Wenqi D Li W Shanshan C Bei C Yafei Z Feihu B Jie L Daiming F EpCAM is overexpressed in gastric cancer and its downregulation suppresses proliferation of gastric cancer. J Cancer Res Clin Oncol 2009 135 1277 85 19294417 10.1007/s00432-009-0569-5 PMC12160224

[30] Crighton D Wilkinson S O'Prey J Syed N Smith P Harrison PR Gasco M Garrone O Crook T Ryan KM DRAM, a p53-induced modulator of autophagy, is critical for apoptosis. Cell 2006 126 121 34 16839881 10.1016/j.cell.2006.05.034

[31] Goldstein JC Muñoz-Pinedo C Ricci JE Adams SR Kelekar A Schuler M Tsien RY Green DR Cytochrome c is released in a single step during apoptosis. Cell Death Differ 2005 12 453 62 15933725 10.1038/sj.cdd.4401596

[32] Baeuerle PA Gires O Ep-CAM (CD326) Finding its role in cancer. Br J Cancer 2007 96 417 23 17211480 10.1038/sj.bjc.6603494 PMC2360029

[33] Orlic M Spencer CE Wang L Gallie BL Expression analysis of 6p22 genomic gain in retinoblastoma. Genes Chromosomes Cancer 2006 45 72 82 16180235 10.1002/gcc.20263

[34] Gladden AB Diehl JA Location, location, location: the role of cyclin D1 nuclear localization in cancer. J Cell Biochem 2005 96 906 13 16163738 10.1002/jcb.20613

[35] Yaari S Jacob-Hirsch J Amariglio N Haklai R Rechavi G Kloog Y Disruption of cooperation between Ras and MycN in human neuroblastoma cells promotes growth arrest. Clin Cancer Res 2005 11 4321 30 15958613 10.1158/1078-0432.CCR-04-2071

[36] MacPherson D Conkrite K Tam M Mukai S Mu D Jacks T Murine bilateral retinoblastoma exhibiting rapid-onset, metastatic progression and N-myc gene amplification. EMBO J 2007 26 784 94 17235288 10.1038/sj.emboj.7601515 PMC1794380

[37] Sakai K Tanooka H Sasaki MS Ejima Y Kaneko A Increase in copy number of N-myc in retinoblastomas in comparison with chromosome abnormality. Cancer Genet Cytogenet 1988 30 119 26 3422037 10.1016/0165-4608(88)90099-4

[38] Adithi M Nalini V Kandalam M Krishnakumar S Expression of matrix metalloproteinases and their inhibitors in retinoblastoma. J Pediatr Hematol Oncol 2007 29 399 405 17551402 10.1097/MPH.0b013e3180683bf1

[39] Adithi M Venkatesan N Kandalam M Biswas J Krishnakumar S Expressions of Rac1, Tiam1 and Cdc42 in retinoblastoma. Exp Eye Res 2006 83 1446 52 17027002 10.1016/j.exer.2006.08.003

[40] Balzar M Winter MJ de Boer CJ Litvinov SV The biology of the 17–1A antigen (Ep-CAM). J Mol Med 1999 77 699 712 10606205 10.1007/s001099900038

[41] Pauli C Münz M Kieu C Mack B Breinl P Wollenberg B Lang S Zeidler R Gires O Tumor-specific glycosylation of the carcinoma associated epithelial cell adhesion molecule Ep-CAM in head and neck carcinomas. Cancer Lett 2003 193 25 32 12691820 10.1016/s0304-3835(03)00003-x

